# Do Gastric Signet Ring Cell Carcinomas and ECL-Cell Neuroendocrine Tumours Have a Common Origin?

**DOI:** 10.3390/medicina58040470

**Published:** 2022-03-23

**Authors:** Reidar Fossmark, Rune Johannessen, Gunnar Qvigstad, Patricia Mjønes

**Affiliations:** 1 Department of Gastroenterology and Hepatology, St. Olav’s Hospital, 7030 Trondheim, Norway; rune.johannessen@stolav.no (R.J.); gunnar.qvigstad@stolav.no (G.Q.); 2 Department of Cancer Research and Molecular Medicine, Faculty of Medicine, Norwegian University of Science and Technology, 7030 Trondheim, Norway; patricia.mjones@stolav.no; 3 Department of Pathology, St. Olav’s Hospital, 7030 Trondheim, Norway

**Keywords:** signet ring cell carcinoma, carcinoid, neuroendocrine tumour, gastrin, carcinogenesis

## Abstract

Gastric cancer is a heterogenous group of tumours, and a better understanding of the carcinogenesis and cellular origin of the various sub-types could affect prevention and future treatment. Gastric neuroendocrine tumours (NETs) and adenocarcinomas that develop in the gastric corpus and fundus of patients with chronic atrophic gastritis have atrophic gastritis, hypoacidity, and hypergastrinemia as common risk factors and a shared cellular origin has been suggested. In particular, signet ring cell carcinomas have previously been suggested to be of neuroendocrine origin. We present a case of a combined gastric NET and signet ring cell carcinoma in a patient with hypergastrinemia due to autoimmune chronic atrophic gastritis. The occurrence of such a combined tumour strengthens the evidence that gastric NETs and signet ring cell carcinomas develop from a common origin.

## 1. Introduction

The proliferation of enterochromaffin-like (ECL) cells of the gastric corpus and fundus is stimulated by gastrin and most gastric neuroendocrine tumours (NETs) develop due to long-term hypergastrinemia. Gastric NETs develop in patients with autoimmune chronic atrophic gastritis (CAG) (type 1), as well as in patients with gastrinomas (type 2), and type 1 gastric NETs regress after treatment with a gastrin receptor antagonist [1,2]. The main risk factor for gastric adenocarcinomas is Helicobacter pylori infection with extensive atrophic gastritis [3] that leads to gastric hypoacidity. The cellular origin of gastric adenocarcinomas of the Laurens diffuse type including signet ring cell carcinomas that develop in patients with CAG has been disputed [4]. It has been suggested that gastric signet ring cell carcinomas have a neuroendocrine origin [5,6,7,8], as a high proportion of the tumours are positive for neuroendocrine markers, such as chromogranin A and synaptophysin. Hypergastrinemia is a risk factor for both gastric NETs and adenocarcinomas in patients with CAG [9] and further efforts to understand the formation of both types of gastric neoplasia are needed.

We here present a case of a combined type 1 gastric NET and signet ring cell carcinoma which offers novel information concerning the aetiology and origin of signet ring cell carcinomas.

## 2. Case Report

**Figure 1:**
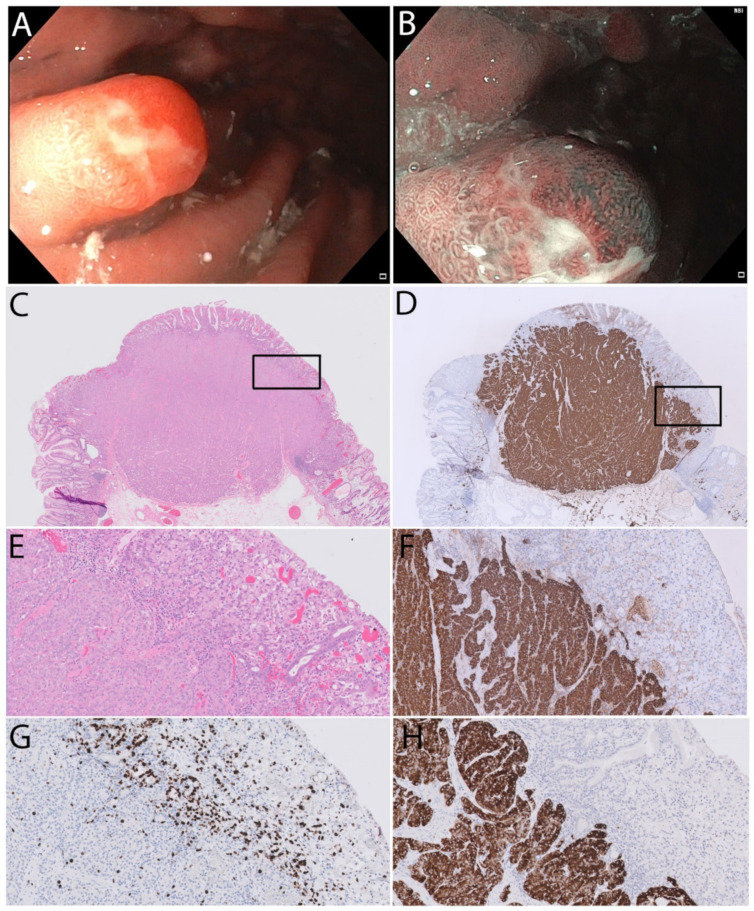
Endoscopic appearance of a polyp located at the major curvature with white light (**A**) and narrow band imaging (**B**). The HE stained sections (**C**,**E**), chromogranin A (**D**,**F**), and synaptophysin (**H**) sections demonstrated well-differentiated neuroendocrine tumour (NET) tissue combined with signet ring cell carcinoma. The proliferation rate evaluated by Ki-67 positivity in the NET component was up to 4% compared to 40% in the signet ring cell component (**G**). The black rectangle in (**C**) is seen at higher magnification as (**E**) and the black rectangle in (**D**) is seen at higher magnification as (**F**). The lower left half of (**E**–**H**) contains NET tissue while the upper right part contains the signet ring cell carcinoma.

An 80-year-old woman with a pervious history of coronary heart disease and pulmonary embolism, using acetylsalicylic acid and warfarin, was referred to our department due to iron deficiency anaemia. After a colonoscopy without relevant pathology, she underwent upper endoscopy. A polyp of 8 mm (Paris Is/Isp) with reddish appearance (Figure 1) located in the gastric corpus along the major curvature was biopsied. Unexpectedly, the biopsies contained atrophic mucosa with an area of 0.5 mm with adenocarcinoma of Laurens diffuse type and signet ring morphology. The polyp was, therefore, removed by endoscopic mucosal resection with a hot snare after elevation with mannitol and indigocarmine. The histology of the resected polyp revealed a combined NET and signet ring cell carcinoma with free lateral and vertical margins. The signet ring component was intramucosal and mainly above the NET, which infiltrated the lamina propria but not the muscularis mucosa. The NET component was chromogranin A (CgA) and synaptophysin positive, whereas the signet ring component was only weakly positive for CgA. The proliferation rate in the NET was 4% in hot-spots and the NET was graded as G2 and the signet ring cell component had a much higher proliferation rate of 40% in hot-spots.

Immunohistochemical methods: 4 µm sections from formalin fixed and paraffin embedded tissue blocks were incubated with antibodies against neuroendocrine markers CgA (M0869, Dako, Glostrup, Denmark, 1:4000) and synaptophysin (M7315, Dako, 1:200). Proliferation rate was assessed in sections labelled with an antibody against Ki-67 (M7240, Dako 1:100). The immunoreaction against synaptophysin was amplified using Mouse Link (K8021, Dako); and, finally, all immunoreactions were visualized using an EnVision-HRP kit with DAB+ (K5007, Dako). Hyperplasia and dysplasia of neuroendocrine cells in the oxyntic mucosa were described according to Solcia [10] in CgA and synaptophysin immunolabelled sections. The proportion of Ki-67 positive cell in “hot spots” was recorded and the tumours graded according to WHO/ENETS [11].

Blood samples included serum gastrin 1226 pM (<60 pM), chromogranin A 243 µg/L (<101.9 µg/L), anti-intrinsic factor > 480 kU/L (<10 kU/L), anti-parietal cell IgG 3 kU/L (<10 kU/L), suggestive of marked hypergastrinemia due to autoimmune chronic atrophic gastritis. Biopsies from the antrum and corpus were H pylori PCR negative and the flat corpus mucosa had changes consistent with autoimmune chronic atrophic gastritis with linear and micronodular neuroendocrine cell hyperplasia [10] and confirmed the diagnosis of a type 1 gastric NET G2. There were no signs of recurrent disease at endoscopic examination at six and 12 months after resection.

## 3. Discussion

The case of a combined gastric NET and signet ring cell carcinoma is a rare occurrence, which we consider an illustrative argument of a common origin of neoplastic lesions with a different morphology. It has been reported that chronic atrophic gastritis and hypergastrinemia predispose to gastric NETs and adenocarcinoma to a similar extent [9] and others have reported the co-existence of multiple NETs and signet ring cell carcinomas in chronic atrophic gastritis [12], suggesting that these tumours have common risk factors. In a large population-based study, we have recently found that hypergastrinemia predisposes to development of adenocarcinomas in the corpus and fundus only [13], where gastrin exerts its trophic effect [14]. Furthermore, we have previously described multiple gastric NETs G1 in a family with hypergastrinemia due to an inactivating mutation in the ATP4A gene encoding the alpha subunit of the gastric H^+^K^+^ATPase, where a combined NET G2 and adenocarcinoma also was found [15,16]. Similarly, in animal models of hypergastrinemia both ECL cell NETs and tumours with adenocarcinoma phenotype develop in the oxyntic mucosa [17].

According to the 2019 WHO classification, a mixed neuroendocrine-non-neuroendocrine neoplasm (MiNEN) has components (typically neuroendocrine and adenocarcinoma components) that each constitute at least 30% [18], a limit that may seem arbitrary. In the current case the rapidly proliferating signet ring cell component was less than 30% at the time of endoscopic resection but could have been the dominant component if examined at a later timepoint. The presented tumour should most likely be classified as a neuroendocrine tumour with a focal non-neuroendocrine component [18,19]. The mentioned aspects illustrate that classifications based on strict cut-off values represent operational definitions useful for some purposes but do not represent an etiological classification as they ignore the dynamic evolution of mixed tumours. A prevailing hypothesis has been that NETs originate from proliferating ECL-cells residing at the base of the gastric glands through a hyperplasia-dysplasia-neoplasia sequence [20], whereas adenocarcinomas develop from stem cells [21] that in the gastric oxyntic glands are located in the isthmus region. The occurrence of tumours where those two components are mixed implies that two different cancers have developed simultaneously from separate cell populations that are normally separated. This is difficult to explain within this paradigm. Such seemingly paradoxical events encourage alternative hypotheses that could explain an observed phenomenon. It has been suggested by several research groups that signet ring cell carcinomas are of neuroendocrine (NE) origin [5,6,7,8]. Germline mutations in CHD1 cause development of diffuse gastric cancers [22] and many sporadic signet ring cell carcinomas also have mutations in CDH1 [23]. CDH1 mutations have not been reported in gastric NETs, however, normal gastric NE cells do not express E-cadherin [24]. We find it less likely that tumours with both NE and signet ring cell components represent collisions of two entirely independent clones, but rather develop from a common origin and that morphological and mutational differences evolve with time.

## 4. Conclusions

Altogether, the current case report describes a rare event but supports the hypothesis that gastric ECL cell NETs and signet ring cell carcinomas share a common origin.

## Data Availability

Not applicable.
